# Fetuin-A as a predicator of sarcopenic left ventricular dysfunction

**DOI:** 10.1038/srep12078

**Published:** 2015-07-10

**Authors:** Wei-Ting Chang, Wei-Chuan Tsai, Chih-Hsing Wu, Yen-Wei Lee, Yun-Lin Tai, Yi-Heng Li, Liang-Miin Tsai, Jyh-Hong Chen, Ping-Yen Liu

**Affiliations:** 1 Division of Cardiology, Internal Medicine, College of Medicine, National Cheng Kung University Hospital, Tainan, Taiwan; 2 Institute of Clinical Medicine, College of Medicine, National Cheng Kung University, Tainan, Taiwan; 3 Division of Cardiovascular Medicine, Chi-Mei Medical Center, Tainan, Taiwan; 4 Department of Family Medicine, College of Medicine, National Cheng Kung University Hospital, Tainan, Taiwan; 5 College of Medicine, National Cheng Kung University, Tainan, Taiwan

## Abstract

Sarcopenia is an aging condition involving low muscle mass and function. Fetuin-A (FetA) appears to be a factor for body composition remodeling. We hypothesized that age increases FetA levels and deteriorates the myocardial function by affecting diastolic function, especially in people with sarcopenia. We enrolled 541 asymptomatic elderly (≥65 years) patients. Compared with non-sarcopenic population, FetA levels were significantly elevated in the ninety-two (17%) patients (79 ± 6 years; male: 34.7%) diagnosed with sarcopenia (621.1 ± 140.7 vs. 697.3 ± 179.6 μg/ml, < 0.001). Sarcopenic left ventricular dysfunction (S-LVD) was defined by the coexistence of sarcopenia and systolic impairment (LVEF < 50%) and 23 (4.3%) of them met the criteria. Patients with S-LVD showed relatively reduced systolic heart function, higher end-diastolic pressure and a higher FetA level (all p < 0.001) than did those with sarcopenia but without LV dysfunction (S-NLVD). Conversely, in the group without sarcopenia, FetA levels were similar regardless of systolic function. Multivariable logistic regression showed that older age, impaired diastolic function, and higher FetA levels were significantly associated with S-LVD. In conclusion, we found that FetA was significantly higher in elderly patients with sarcopenia, which was associated with impaired diastolic and systolic functions.

Sarcopenia is defined as age-related muscle loss characterized by the involuntary reduction in fatty-free muscle mass, strength, and function^1^,^2^. Documented in skeletal muscle and aortic smooth muscle, sarcopenia is associated with a decline in protein synthesis, which causes a decrease in type II fibers and in myosin heavy chains^3^,^4^,^5^. Although age-related cardiac changes have been well described in humans and rats^6^,^7^, only few literatures determined whether sarcopenia occurs in cardiac muscle^8^,^9^,^10^. Sarcopenia has been recently correlated with several degenerative processes. Some hypotheses have linked it to insulin-like growth factor-1 (IGF-1), nuclear factor kappa-light-chain-enhancer of activated B cells (NF-κB), and apoptosis^3^,^4^. Notably, in the aging heart an increasing incidence of heart failure has been observed in the elderly (≥65 years), especially those with sarcopenia^6^,^8^,^9^,^11^.

Fetuin-A (FetA), a glycoprotein synthesized in the liver and secreted into the circulation, is a calcification inhibitor as well as a conjunction in insulin resistance^12^,^13^,^14^,^15^,^16^. It was recently reported to be a main factor in adjusting body composition remodeling in the geriatric population^17^. Emerging evidence associates FetA with promoting cardiovascular disease by causing coronary and valvular calcification^18^,^19^. In addition, higher FetA levels were found to correlate with poor cardiovascular outcomes, including myocardial infarction and ischemic stroke through the insulin related NF-κB axis^20^,^21^. However, whether increased FetA exacerbates the progression of cardiac dysfunction remains unknown. Therefore, we hypothesized that age increases FetA levels and deteriorates the myocardial function by affecting global cardiac function, especially in people with sarcopenia.

## Materials and Methods

### Study Population

Tianliao Township is a suburban community in Kaohsiung County in southern Taiwan. Almost a quarter (23.7%) of the 31,000 residents are older than 65 y/o. The Tianliao Old People (TOP) study^22^ has been conducted in an aged cohort since 2009 in order to develop better community-oriented primary care services. According to the 2010 census, there were 1,033 elderly men in Tianliao. We did an epidemiological survey using the whole community sampling method in July 2010. After excluding empty houses (n = 269), deaths (n = 21), and non-ambulatory residents (n = 62), only 681 residents were eligible, and 541 residents were enrolled. The response rate was 60.8% and the statistical power was 0.80. This study was approved by the Institutional Review Board of our institute (IRB no: ER-99-111), the methods were carried out in accordance with the approved guidelines. Also, each participant signed the informed consent before they were examined. Anthropometric (e.g., body mass index (BMI)) and medical information, including previous strokes, coronary artery disease, arrhythmia, and cancer, was collected using a questionnaire. Duplicate waist circumference measurements were done on bare skin midway between the lower rib margin and the iliac crest at the end of normal expiration. Diabetes mellitus was defined according to American Diabetes Association 2010 diagnostic criteria and the medical history reported by each participant.

### Laboratory Methods

After the participants had fasted for at least 8 hours, peripheral blood samples were collected from them and centrifuged at 3,000 rpm for 15 minutes at 4 °C. The samples were frozen and sent to a central laboratory for analysis. Serum FetA was determined by an ELISA method (intraassay CV of 2.7%, interassay CV of 3.2%; Biovendor Laboratory Medicine, Brno, Czech Republic). The minimum detectable dose of FetA is 9.38 ng/ml.

### Definition of Sarcopenia

To define sarcopenia in this cohort study, we measured skeletal muscle mass (SMM) using a bioelectrical resistance system (BioScan 920; Maltron, Rayleigh, Essex, UK), a convenient and radio-free tool with an operating frequency of 50 kHz at 800 mA. Participants were supine on a non-conducting surface with their arms abducted away from their trunk and their legs slightly separated for 5 minutes. Four electrodes and cables were attached to their right hand and ankle. When the measurements stabilized, the analyzer displayed resistance directly and immediately. SMM was calculated using the BIA equation described elsewhere^2^:

- SMM = [(Ht^2^/*R* × 0.401) + (gender × 3.825) + (age × −0.071)] + 5.102
- Skeletal muscle index (SMI) = SMM/Ht^2^

For the associated units: height in cm; resistance in ohms; gender (51 men, 50 women); and age in years. Absolute SMM was converted to an SMI by dividing it by height in square meters (SMM/Ht^2^). Based on previous reference, the normal range of the SMI was defined as above 7.23 for men and above 5.67 for women^22^.

Sarcopenia was defined using the criteria of the European Working Group on Sarcopenia in Older People (EWGSOP)^2^ in 2010 as presenting 3 of the following components: (1) loss of muscle mass: an SMI of ≥2 standard deviations (SDs) less than the normal gender-specific mean. In the studied population, an SMI <7.23 in men and 5.67 in women meant being assigned to the sarcopenic group; (2) low muscle strength: the maximal weight of hand-grasping <30 pounds in men and <20 pounds in women; (3) low physical performance: a maximal walking speed <0.8 m/s.

### Echocardiography

Standard echocardiography was done (Vivid I; GE Vingmed Ultrasound AS, Horten, Norway) using a 3.5-MHz multiphase-array probe based on the recommendations of the American Society of Echocardiography^23^. The chamber dimensions and left ventricular (LV) mass were measured using the two-dimensionally guided M-mode method, and the LV ejection fraction (LVEF) was measured using the two-dimensional M mode. Transmitral Doppler flow velocity was obtained from an apical four-chamber view, and peak early filling velocity (E), peak atrial velocity (A), and the E/A ratio were recorded. Early mitral inflow velocity (E) and early annular diastolic velocity (E’) were also measured to estimate the LV end-diastolic pressure (E/E’). Both the medial and lateral early annular diastolic velocity values were measured to achieve the average value. Echocardiographic diastolic dysfunction was defined as the coexistence of impaired relaxation (E’ < 8 cm/s by tissue Doppler) and elevated average LV end-diastolic pressure (E/E’ > 8)^24^. To distinguish from the population with preserved LV systolic function, subjects with LVEF below 50% were defined as impaired LV systolic function or LVD^25^. The systolic and diastolic function of each echocardiographic imaging was analyzed by experienced cardiologists triplicately. Participants who fulfilled both the criteria of sarcopenia and of impaired LV systolic function were assigned to the sarcopenic LV dysfunction (S-LVD) group; participants with sarcopenia and preserved systolic function (LVEF ≥ 50%) were assigned to the Sarcopenic-non LV dysfunction group (S-NLVD); those without sarcopenia but with impaired systolic function were assigned to the NS-LVD group; and, finally, those without sarcopenia and with preserved systolic function were assigned to the NS-NLVD group.

### Statistical Analysis

Data management and statistical analyses were done using SPSS 18.0 (SPSS Inc., Chicago, IL). Continuous variables with a normal distribution (fasting blood sugar, total cholesterol, HDL-C, LDL-C, FetA, waist circumference, height, weight, body fat, and BMI) are expressed as means ± standard deviation (SD). Other continuous variables with a non-normal distribution (age, hs-CRP level, triglycerides) are expressed as means ± standard error (SE). Continuous variables were compared using a Student’s *t* test for normally distributed values. Comparisons between categorical variables were made using a κ^2^ test. A Spearman partial correlation coefficient between FetA and LVEF was calculated. To validate the association between sarcopenia and heart function, we separated the participants into sarcopenia as well as non-sarcopenia groups and then further classified each based on whether or not LVEF was >50%. Group differences were analyzed using analysis of variance (ANOVA). Significant differences verified using a Tukey post hoc test in single variant analysis were entered into multivariate analysis. Odds ratios (ORs) and 95% confidence intervals (CIs) were assessed using logistic regression. Statistical tests were 2-sided; significance was set at *p* < 0.05. Receiver operating characteristic (ROC) curve analysis was used to determine the optimal cutoff values of FetA in patients with and without sarcopenic cardiomyopathy. The best cutoff value was defined as the point with the highest sum of sensitivity and specificity.

## Results

### Clinical and Echocardiographic Differences between Subpopulations with and without Sarcopenia

Ninety-two (17%, mean age: 79 ± 6 years; male: n = 32 (34.7%)) of our 541 patients (mean age: 75 ± 6 years; male: n = 283 (52.3%)) were diagnosed with sarcopenia (Table 1). None of them was reported with specific heart failure symptoms. They were significantly older, more were female, had a smaller waist circumference (WC), a lower BMI, and a lower average blood pressure (BP) (all *p* = 0.001). Echocardiography showed preserved E/E’. Their serologic profiles were similar, except for significantly higher HDL-C (*p* = 0.003) and FetA levels (*p* < 0.001) for patients with sarcopenia.

**Figure 1:**
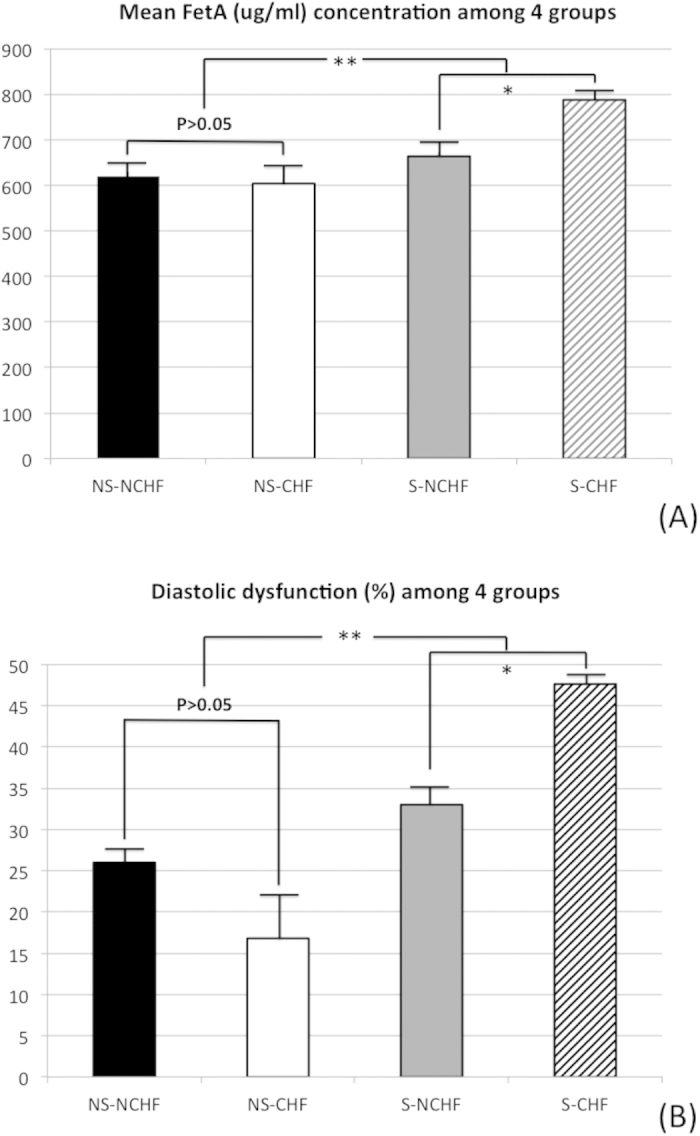
Higher level of fetuin-A (FetA) was associated with sarcopenic subjects, especially with systolic dysfunction. (**A**) serum levels of FetA (μg/mL) and (**B**) diastolic dysfunction (E′ < 8 cm/s and E/E′ > 8) prevalence rate in the groups without sarcopenia and left ventricular systolic dysfunction (NS-NLVD), without sarcopenia but with left ventricular systolic dysfunction (NS-LVD), with sarcopenia but with non- left ventricular systolic dysfunction (S-NLVD), and with sarcopenia and left ventricular systolic dysfunction (S-LVD). Abbreviations: FetA, fetuin-A; E/E′, ratio of early transmitral flow velocity (E) to early diastolic mitral annulus velocity (e′). *p < 0.001; **p < 0.01.

In this cohort, 23 (25%) of the 92 patients with sarcopenia met the S-LVD criteria (Table 2). Significantly more patients in the S-LVD subgroup were male (*p* < 0.001) and had stroke, coronary artery disease (CAD), and arrhythmia prevalence rates that were not significantly different. Echocardiography showed a non-significantly higher left ventricular mass index (LVMI) and a significantly higher estimated end-diastolic pressure (E/E′) in the S-LVD subgroup, which might indicate stiffness of the myocardium or diastolic dysfunction. The serologic profiles showed significantly higher HDL-C (*p* = 0.034) and FetA (*p* < 0.001) levels in the S-LVD subgroup patients. They had the highest E/E′, FetA levels and the lowest E′ of the 4 subgroups. In the group with sarcopenia, FetA was significantly (*p* < 0.001) higher in the S-LVD group (788.2 ± 170.2 μg/ml) than in the S-NLVD group (664.3 ± 163.8), but the difference of FetA levels was not significant in the non-sarcopenia group (Fig. 1A).

Our echocardiographic analysis of diastolic function used impaired relaxation (E’ < 8 cm/s by tissue Doppler) and subsequently developed high left ventricular end-diastolic pressure (E/E’ > 8) to facilitate the definite diagnosis. Similar to systolic dysfunction, the prevalence of diastolic dysfunction was significantly (*p* = 0.001) higher in the sarcopenia group and highest in the S-LVD subgroup, but the difference between the NS-LVD and NS-NLVD subgroups was not significant (Fig. 1B). This indicates the different effects of FetA on myocardium contractility and relaxation functions in the elderly with and without sarcopenia.

Ischemic cardiomyopathy is a known major predisposing factor for geriatric cardiac dysfunction; however, in our study, by comparing participants with (n = 13) and without cardiovascular diseases (n = 258), the FetA concentrations were not significantly different (652.6 ± 159.4 and 630.9 ± 149.6 mg/L, respectively; *p* = 0.34).

### Characteristics of FetA in the Geriatric Population

**Figure 2:**
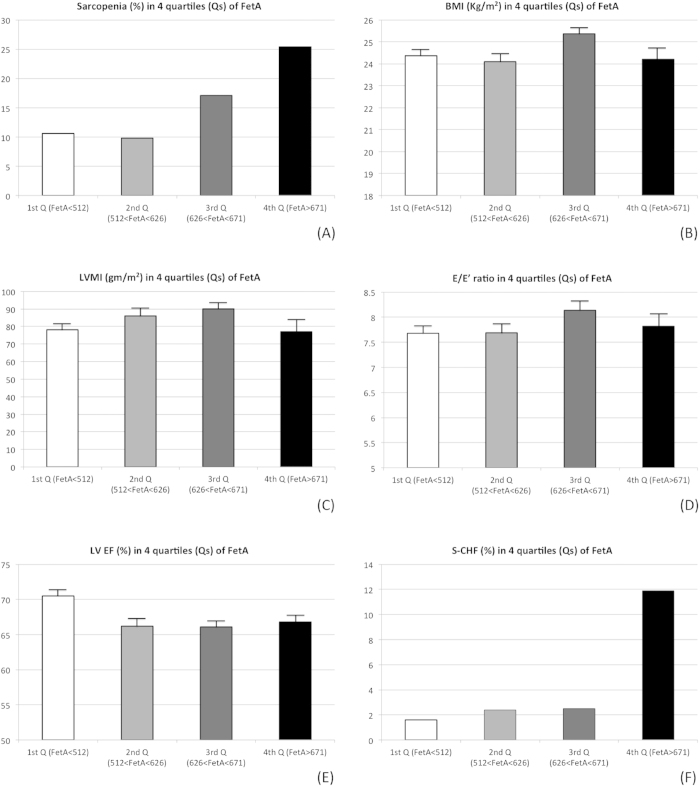
Incrementing FetA quartiles positively correlated with sarcopenia, especially with sarcopenic left ventricular systolic dysfunction (S-LVD). (**A**) sarcopenia; (**B**) body mass index (BMI) level; (**C**) left ventricular mass index (LVMI) (by echocardiography); (**D**) diastolic dysfunction; (**E**) systolic dysfunction; and (**F**) S-LVD. *p < 0.001; **p < 0.01.

**Figure 3:**
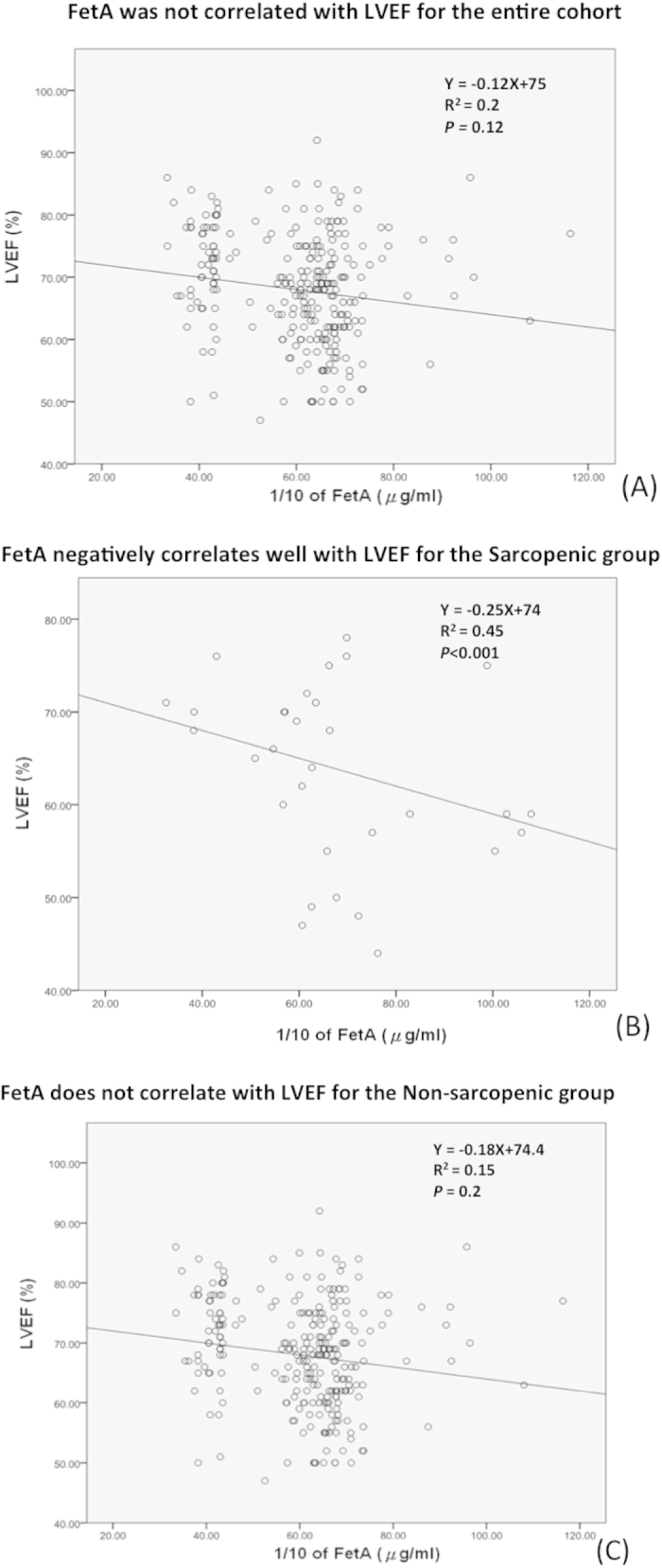
Fetuin-A (FetA) serum concentrations negatively correlated with left ventricular ejection faction (LVEF), significantly in the sarcopenic population. (**A**) all geriatric participants; (**B**) in the sarcopenic sub-population and (**C**) in the non-sarcopenic subpopulation.

In this geriatric population, the mean value of serum FetA levels was 633.0 mg/L, which was not different by gender (626.1 ± 149.5; 640.5 ± 150.7 mg/L, respectively; *p* = 0.19) (data not shown). To test the pathophysiologic role of FetA in sarcopenia and heart function, we compared 4 subgroups using the quartiles of FetA (Fig. 2A–F). With a similar disease background, the prevalence of sarcopenia and S-LVD increased with incrementing FetA quartiles. To compare the interplay between FetA and heart function, the association between FetA level and LVEF was echocardiographically analyzed. Interestingly, although the LVEF value was not strongly correlated with the increased quartiles of FetA in the entire study population, the LVEF value was significantly negatively correlated with the FetA level in the sarcopenic group (R^2^ = 0.45, *p* < 0.001), but not in the non-sarcopenic group (R^2^ = 0.15, *p* = 0.2) (Fig. 3A–C), which implied the importance of the co-existence of sarcopenia in the link between FetA and LV dysfunction.

**Figure 4:**
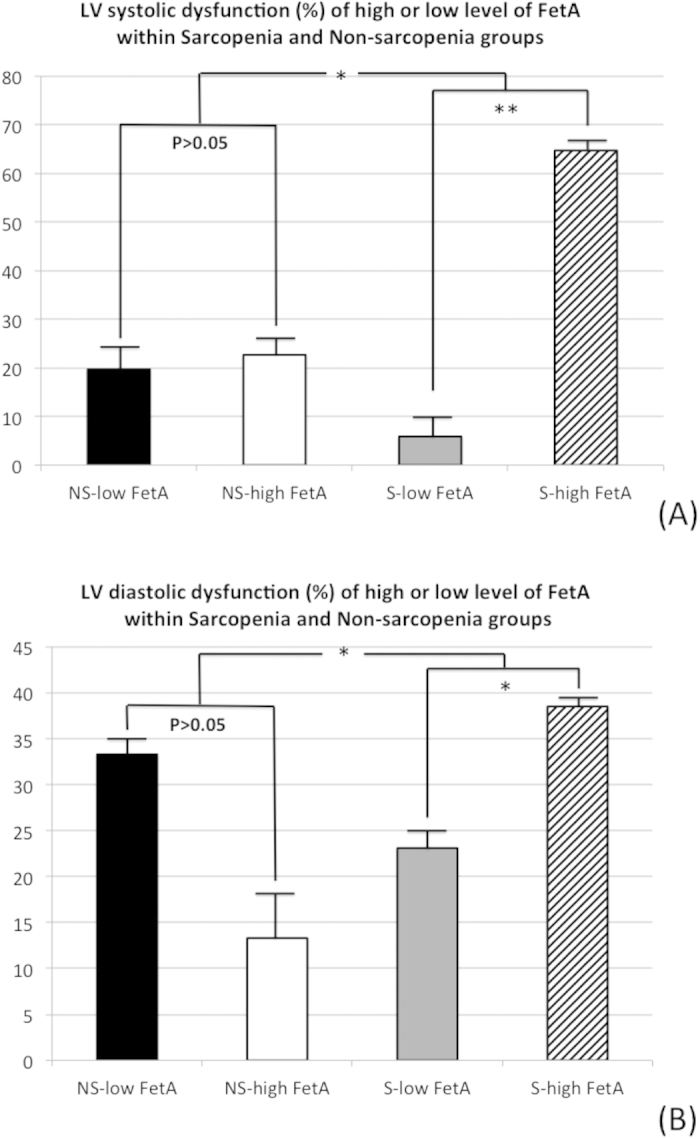
The percentages of left ventricular systolic and diastolic dysfunction were significantly higher in sarcopenic elders with higher fetuin-A (FetA) level. (**A**) left ventricular (LV) systolic impairment and (**B**) LV diastolic dysfunction in the non-sarcopenia and sarcopenia sub-populations according to the higher and lower FetA level. Lower FetA: FetA below the 1st quartile; higher FetA: FetA above the 3rd quartile; LV systolic dysfunction: left ventricular ejection faction (LVEF) <50%; LV diastolic dysfunction: coexistence of impaired relaxation (Ea < 8 cm/s by tissue Doppler) and elevated left ventricular end-diastolic pressure (E/Ea > 8) from echocardiography. *p < 0.001; **p < 0.01.

To determine whether FetA levels are different in the elderly with and without sarcopenia, the percentages of “higher FetA level” (>75% 3rd quartile of the whole population) and “lower FetA level” (<25% 1st quartile of the whole population) in relationship to LV systolic and diastolic dysfunction were analyzed (Fig. 4A). The prevalence rates of high and low FetA levels were not significantly different for the non-sarcopenia subgroups (53.4% vs. 46.6%, *p* > 0.05), but FetA was significantly higher for patients with sarcopenia (71.1% vs. 28.9%, *p* >0.05). Moreover, in the non-sarcopenia group, both the high and low FetA levels were associated with the similar percentages of LV systolic dysfunction (22.7% vs. 19.7%, *p* >0.05). Interestingly, in the sarcopenia group, those with a high FetA level were more significantly associated with higher incidence of LV systolic dysfunction than were those with a low FetA level (64.7% vs. 5.9%, *p* <0.05). The percentages of diastolic function as well as high and low FetA levels were similar to those in patients with systolic impairment (Fig. 4B). All the results for this analysis implied that the specific role of sarcopenia in FetA is related to the impairment of both systolic and diastolic heart function.

### Diagnostic Value of FetA in Sarcopenia and Sarcopenic Left Ventricular Dysfunction

S-LVD was associated with multiple risk factors. A multivariable logistic regression test to determine the independent risk factors showed that, in this cohort, in addition to the known risk factors for sarcopenia, e.g., a reduced waist circumference and a high E/E′ value (Table 2), a high FetA level was an independent risk factor for S-LVD (Table 3).

**Figure 5:**
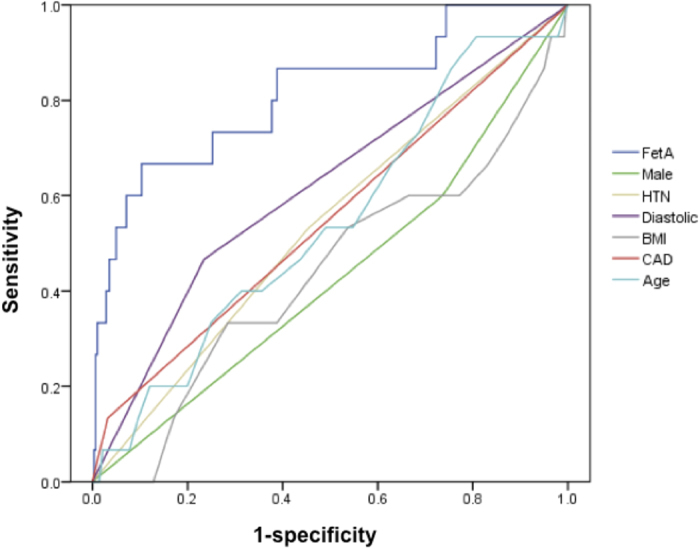
Power of fetuin-A (FetA) to predict sarcopenia with left ventricular systolic dysfunction. The area under the receiver operating characteristic curves was 0.81 for FetA, 0.61 for diastolic dysfunction, 0.53 for hypertension (HTN), 0.61 for coronary artery disease (CAD), 0.44 for body mass index (BMI), 0.53 for age, and 0.43 for male gender.

The area under the ROC curve was 0.81 for S-LVD (Fig. 5). For predicting S-LVD, using FetA >671 mg/L as a cutoff point, the sensitivity was 65.2% and the specificity was 76.4%. Similarly, a low FetA level (<512 mg/L) implied a less probability of S-LVD (true negative: 92.3%).

## Discussion

In this study, we investigated the effects of sarcopenia, a systemic aging process that seems to present not only the wasting of skeletal muscle, but also of the myocardium. We indicated a new term that describes comorbid sarcopenia and systolic heart dysfunction: “sarcopenic LV dysfunction (S-LVD)”. We found that sarcopenic patients were prone to a relatively reduced systolic heart function, increased end-diastolic pressure and a higher FetA level.

Compared with other Asian geriatric studies, we found a relatively lower prevalence of sarcopenia^6^. According to the literature, muscle strength decline is typically observed in 20–40% of older people by their sixties and in more people over 70 years old^1^,^2^. The estimated prevalence of sarcopenia in older men and women worldwide varies from 3% to 30%, and even approached 50% in those over 75 years old^2^. Our survey data showed a 17% prevalence rate of sarcopenia in this Asian geriatric community. One possible reason might be the ethnic variation between Caucasian and Asian population. The other explanation for the discrepancy in this study may be attributable to the rural agricultural lifestyle lived by most residents of Tianliao Township: they are more physically active than most urban residents. Regarding the biomarkers evaluation, people over 65 had lower albumin levels as well as less insulin resistance and dyslipidemia. Interestingly, in the echocardiographic study, they had a higher E/E′. These results highlighted the effect of aging not only presented in skeletal muscle, but also in nutrition, kidney as well as the cardiac function.

More recently, we also documented stiffer characteristic hearts over the elderly population than the younger’s hearts^26^. Age-associated cellular and molecular mechanisms involve cardiomyocyte function, especially apoptosis, alterations of neurohormonal regulation, and changes in the extracellular matrix^27^. In an aging heart animal model, intracellular lipid accumulation and a decrease in glycogen storage were both found in senescent mice^7^. A mathematical simulation also indicated that several changes in LV dimension, collagen deposition, wall stress, and wall stiffness precede LV dysfunction^7^. In humans, many mechanisms have been proposed for the aging process, including mitochondrial function changes, senescence adaption, and telomerase shortening^28^. Quantitative studies have shown that a continuous loss of myocytes is accompanied by reactive hypertrophy of the remaining cells in the aging heart^7^,^29^.

In the present study, we indicated that an increase in the serum FetA level was associated with gradually declined heart function. Also, FetA was specifically elevated in the sarcopenic population with impaired LVEF, which was absent in the non-sarcopenic population. The function of FetA in human beings remains controversial, especially with the respect to sarcopenic hearts. FetA, previously recognized as a calcification inhibitor, has been discovered to play different roles in various fields. Despite the reasonable suspicion of its involvement in diastolic dysfunction, FetA knockout mice showed significant myocardial stiffness, cardiac remodeling, and diastolic dysfunction^13^,^18^,^30^. Conversely, overexpression of FetA prevented ectopic mineralization of connective tissues in a mouse model^12^. Notably, sarcopenia was correlated with chronic inflammation. Higher expression of IGF-1 and NF-κB frequently presented in sarcopenic patients^3^,^4^. FetA acts as both a calcium inhibitor and a conjunction of fat related inflammation^12^,^13^,^14^,^15^,^16^,^21^. One recent study reported the association of higher FetA levels not only with a higher incidence of subclinical inflammation, metabolic syndrome, and coronary artery calcification, but also with poor outcomes in myocardial infarction and acute ischemic stroke^21^. Therefore, the behavior of FetA may vary in specific populations, like sarcopenic elders.

In our study, compared with the non-sarcopenia group, the sarcopenic group tended to have a higher concentration of FetA, which was associated with a relatively lower LVEF. This implied a possible link between the activation of FetA and sarcopenia-related systolic as well as diastolic dysfunction. Also, by comparing participants with and without CAD, the averaged FetA levels were similar. The similar levels of FetA between these two groups implied that the changes of FetA probably derived from the aging heart instead of through the process of coronary artery disease. However, whether the increased level of FetA was the reason contributing to the myocardial stiffness or it was the result of a compensatory feedback to relieve the rigidity remains unknown. Further investigations are required to identify the character of FetA in the aging heart.

Our study has some limitations. The older participants may hardly reflect the general population and cardiac dysfunction was defined by echocardiographic parameters while clinical symptoms were lacking.

## Conclusion

FetA expression was significantly higher in the elderly Asian population with sarcopenia, especially when their heart diastolic and systolic functions were impaired.

## Additional Information

**How to cite this article**: Chang, W.-T. *et al.* Fetuin-A as a predicator of sarcopenic left ventricular dysfunction. *Sci. Rep.* **5**, 12078; doi: 10.1038/srep12078 (2015).

## Figures and Tables

**Table 1:** Clinical and echocardiographic characteristics: comparison between Non-Sarcopenia and Sarcopenia groups.

	Non-Sarcopenia (n = 449, 82.9%)	Sarcopenia (n = 92, 17%)	*p*-value
Age (years)	75 ± 5	79 ± 6	0.001*
Male (%)	251 (55.9)	32 (34.7)	0.001*
WC (cm)	88.63 ± 9.77	78.73 ± 8.74	0.001*
BMI (kg/m^2^)	25.3 ± 3.9	22.37 ± 3.1	0.001*
SBP (mmHg)	135.6 ± 20.8	128.5 ± 19.2	0.003*
DBP (mmHg)	77.7 ± 11.7	73.6 ± 11.3	0.002*
Medical History (%)			
CKD	76 (16.9)	45 (48.9)	0.001*
Previous stroke	17 (3.7)	3 (3.2)	0.89
CAD	11 (2.4)	2 (2.2)	0.8
Arrhythmia	19 (4.1)	2 (1.6)	0.8
Cancer	11 (2.4)	4 (3.3)	0.37
Serology profiles			
HbA_1_c (%)	6.1 ± 0.9	6.1 ± 1.1	0.79
TG (mg/dl)	128.6 ± 79.5	113.8 ± 58.2	0.1
Cholesterol (mg/dl)	201.3 ± 36.7	204.1 ± 34.6	0.52
HDL-C (mg/dl)	49.8 ± 12.4	54.4 ± 15.4	0.003*
Total bilirubin (mg/dl)	0.63 ± 0.3	0.61 ± 0.55	0.67
FetA (μg/ml)	621.1 ± 140.7	697.3 ± 179.6	0.0001*
Echocardiographic parameters			
LVMI (g/M^2^)	81.9 ± 20.4	84.2 ± 30.2	0.65
LVEF (%)	68.79 ± 8.6	62.64 ± 9.3	0.007*
E/A	0.79 ± 0.23	0.67 ± 0.32	0.14
E′ (m/s)	0.09 ± 0.03	0.075 ± 0.02	0.08
E/E′	7.5 ± 2.1	9.2 ± 4.4	0.001*

Data are n (%) or mean ± standard error. BMI = body mass index; BW = body weight; CAD = coronary artery disease; CKD = chronic kidney disease (defined by eGFR ≤60 ml/min/1.73 m^2^); DBP = diastolic blood pressure; E/A = peak early filling velocity (E) to peak atrial velocity (A) ratio; E/E’ = early mitral inflow velocity to early annular diastolic velocity ratio; FetA = fetuin-A; HbA_1_C = glycated hemoglobin; HDL-C = high-density lipoprotein cholesterol; LVEF = left ventricular ejection fraction; LVMI = left ventricular mass index; SBP = systolic blood pressure; TG = triglycerides; WC = waist circumference.

*p < 0.05.

**Table 2:** Clinical and echocardiographic characteristics: comparison between non-sarcopenic non- left ventricular systolic dysfunction (NS-NLVD), non-sarcopenic left ventricular systolic dysfunction (NS-LVD), sarcopenic non- left ventricular systolic dysfunction (S-NLVD), sarcopenic left ventricular systolic dysfunction (S-LVD) groups.

	Non-Sarcopenia (NS) (449/541, 82.9%)		Sarcopenia (S) (92/541, 17.0%)		*p*-value
	NS-NLVD (n = 442, 81.7%)	NS-LVD (n = 7, 1.3%)	S-NLVD (n = 69, 12.8%)	S-LVD (n = 23, 4.2%)	
Age (years)	75 ± 6	80 ± 6	80 ± 6	79 ± 6	<0.001*
Male (%)	250 (56.6)	1 (14.3)	21 (30.4)	11 (47.8)	<0.001*
WC (cm)	88.3 ± 9.9	93.5 ± 7.8	78.7 ± 8.7	79.0 ± 7.9	<0.001*
BMI (kg/m^2^)	25.2 ± 4.0	26.9 ± 2.9	21.9 ± 2.9	23.8 ± 4.2	<0.001*
SBP (mmHg)	135.7 ± 21.3	131.4 ± 11.9	127.0 ± 17.8	134.4 ± 15.8	0.014*
DBP (mmHg)	77.7 ± 11.9	74.3 ± 9.7	73.1 ± 10.7	77.5 ± 11.4	0.027*
CKD	80 (18)	2 (25)	28 (43)	11 (52)	0.001*
Stroke	18 (4)	0 (0)	2 (2.9)	0 (0)	0.95
CAD	10 (2.2)	1 (12.5)	2 (2)	0 (0)	0.24
Arrhythmia	17 (3.8)	1 (14.3)	2 (2.9)	0 (0)	0.36
Cancer	10 (2.2)	0 (0)	2 (2.9)	2 (8.7)	0.006*
HbA_1_c (%)	6.1 ± 0.9	5.7 ± 0.1	6.1 ± 1.1	5.9 ± 0.5	0.41
TG (mg/dl)	127.2 ± 78.8	142.1 ± 83.5	127.8 ± 69.4	95.3 ± 37.2	0.28
Cholesterol (mg/dl)	201.5 ± 36.9	190.5 ± 36.1	205.5 ± 34.1	201.1 ± 31.1	0.69
HDL-C (mg/dl)	49.9 ± 12.3	48.4 ± 10.8	53.3 ± 16.6	56.4 ± 13.9	0.034*
Total bilirubin (mg/dl)	0.62 ± 0.27	0.58 ± 0.25	0.64 ± 0.41	0.62 ± 0.48	0.4
FetA (μg/ml)	620.5 ± 142.2	604.9 ± 104.3	664.3 ± 163.8	788.2 ± 170.2	<0.001*
LVMI (g/M^2^)	81.4 ± 19.9	94.4 ± 18.2	81.4 ± 19.7	104.2 ± 45.1	0.073
LVEF (%)	69.1 ± 7.9	49.6 ± 1.1	68.7 ± 8.2	44.5 ± 5.9	<0.001*
E/A	0.8 ± 0.31	0.6 ± 0.2	0.7 ± 0.4	0.6 ± 0.11	0.07
E′ (m/s)	0.09 ± 0.03	0.07 ± 0.02	0.08 ± 0.02	0.06 ± 0.03	0.02*
E/E′	7.6 ± 2.1	8.6 ± 4.6	8.2 ± 2.4	11.4 ± 4.3	<0.001*

Data are n (%) or mean ± standard error. For other abbreviations, see Table 1.

*p < 0.05.

**Table 3:** Multivariate analysis predictors for sarcopenia or sarcopenic left ventricular systolic dysfunction by logistic regression.

Parameter*	Odds Ratio	95% CI	*p*-value
Sarcopenia			
Diastolic dysfunction	4.06	1.62–10.15	0.003*
FetA	1.83	1.02–2.55	<0.001*
Age	1.13	1.09–1.00	<0.001*
SBP	0.98	0.97–0.99	0.003*
DBP	0.97	0.94–0.98	0.003*
LVEF	0.95	0.91–0.98	0.008*
BMI	0.70	0.65–0.77	<0.001*
Male	0.34	0.21–0.5	<0.001*
CAD	0.95	0.2–4.3	0.94
Sarcopenic Heart Failure			
FetA	1.71	1.02–2.31	<0.001*
Age	1.07	1.01–1.14	0.02*
Diastolic dysfunction	2.81	0.98–8.02	0.05*
BMI	0.92	0.82–1.04	0.19
SBP	1.0	0.97–1.03	0.83
DBP	1.02	0.97–1.07	0.52
Male	0.80	0.34–1.86	0.6

Univariates with *p *≤ 0.15 were recruited to multivariate logistic regression analysis using stepwise mode. (LV dysfunction: LVEF < 50%) CI: confidence interval. For other abbreviations, see Table 1. *p < 0.05.
